# AI-generated medical realism and hantavirus misinformation: a new public health threat?

**DOI:** 10.3389/fdgth.2026.1887820

**Published:** 2026-09-10

**Authors:** Marta Colaneri, Alice Baratelli, Sara Laura Ferrari, Juliana Viazzo, Andrea Gori, Simone Di Stefano, Alberto Rizzo, Fabio Borgonovo, Giovanni Scaglione, Stefano Odelli

**Affiliations:** 1 Department of Clinical and Biomedical Sciences, University of Milan, Milan, Italy; 2 Department of Infectious Diseases, “Luigi Sacco” University Hospital, Regional Center for Infectious Diseases (CeReMI), Milan, Italy; 3 Centre for Multidisciplinary Action for Global Health (MAGH), Department of Biomedical and Clinical Sciences, University of Milan, Milan, Italy; 4 Department of Food, Environmental and Nutritional Sciences (DeFENS), University of Milan, Milan, Italy; 5 Laboratory of Clinical Microbiology, Virology, and Bioemergencies, “Luigi Sacco” University Hospital, Regional Center for Infectious Diseases (CeReMI), Milan, Italy; 6 Medical Direction Unit, “Luigi Sacco” University Hospital, Milan, Italy; 7 National PhD Programme in One Health Approaches to Infectious Diseases and Life Science Research, Department of Public Health, Experimental and Forensic Medicine, University of Pavia, Pavia, Italy

**Keywords:** AI-generated medical images, Andes virus, artificial intelligence, hantavirus, misinformation, pandemic preparedness

## Abstract

The widespread availability of generative artificial intelligence (AI) has substantially reshaped the health-information environment compared with the COVID-19 pandemic. Contemporary AI tools can rapidly generate highly realistic medical-looking content from text, including laboratory reports, radiological images, histological slides, and institutional clinical documentation. During infectious disease outbreaks, such simulated medical content may exploit the visual authority traditionally associated with healthcare institutions and influence public risk perception. During media coverage of the May 2026 Andes virus (ANDV) alert in Italy, news outlets published realistic AI-generated medical imagery depicting hantavirus diagnostic reports, hospital-associated laboratory materials, and outbreak-response scenes linked to real healthcare institutions. One image, in particular, reproduced the visual identity of a real laboratory report produced at “Luigi Sacco” University Hospital despite displaying multiple inconsistencies. In some additional examples, AI generation was not clearly disclosed despite the use of recognizable institutional names and logos. This phenomenon has the potential to extend beyond isolated misinformation events. Emerging evidence indicates that experts may struggle to reliably distinguish AI-generated biomedical and radiological images from authentic materials, raising concern that future infectious disease outbreaks may occur within an information ecosystem increasingly shaped by visually persuasive synthetic media. Such content may amplify public anxiety, distort perceptions of authority, and erode trust in institutions. Synthetic medical realism should therefore be considered a potential emerging category of public-health risk during infectious disease emergencies that requires coordinated action from institutions and regulators developing verification systems, while news media, platforms, and publishers implement AI-disclosure standards and guidelines for synthetic biomedical content.

## Introduction

Compared to the COVID-19 pandemic, the current information landscape is being profoundly reshaped by the widespread availability of generative artificial intelligence (genAI).

Large language models (LLMs), multimodal AI systems, and generative image platforms are now increasingly integrated into healthcare communication, scientific dissemination, clinical documentation, and medical education (1). At the same time, concerns regarding the misuse of genAI in medicine are rapidly emerging. Recent literature has documented multiple real-world incidents involving AI-generated misinformation, hallucinated medical content, unsafe clinical suggestions, biased algorithmic outputs, and misleading patient-facing communication (2, 3).

The growing accessibility of genAI tools means that highly realistic medical-looking content can now be produced by virtually anyone within minutes, including radiological images, histological slides, microbiological results, and clinical documentation (4–6). This results in “synthetic medical realism,” defined as multimodal AI-generated content that simulates authentic clinical, laboratory, or institutional medical materials. Unlike during the COVID-19 pandemic, when misinformation spread primarily through fabricated narratives, edited videos, screenshots, and decontextualized media (7), contemporary AI tools can generate synthetic medical artifacts that closely reproduce the visual language and institutional aesthetics of authentic healthcare documentation (8).

## The case

**Figure 1:**
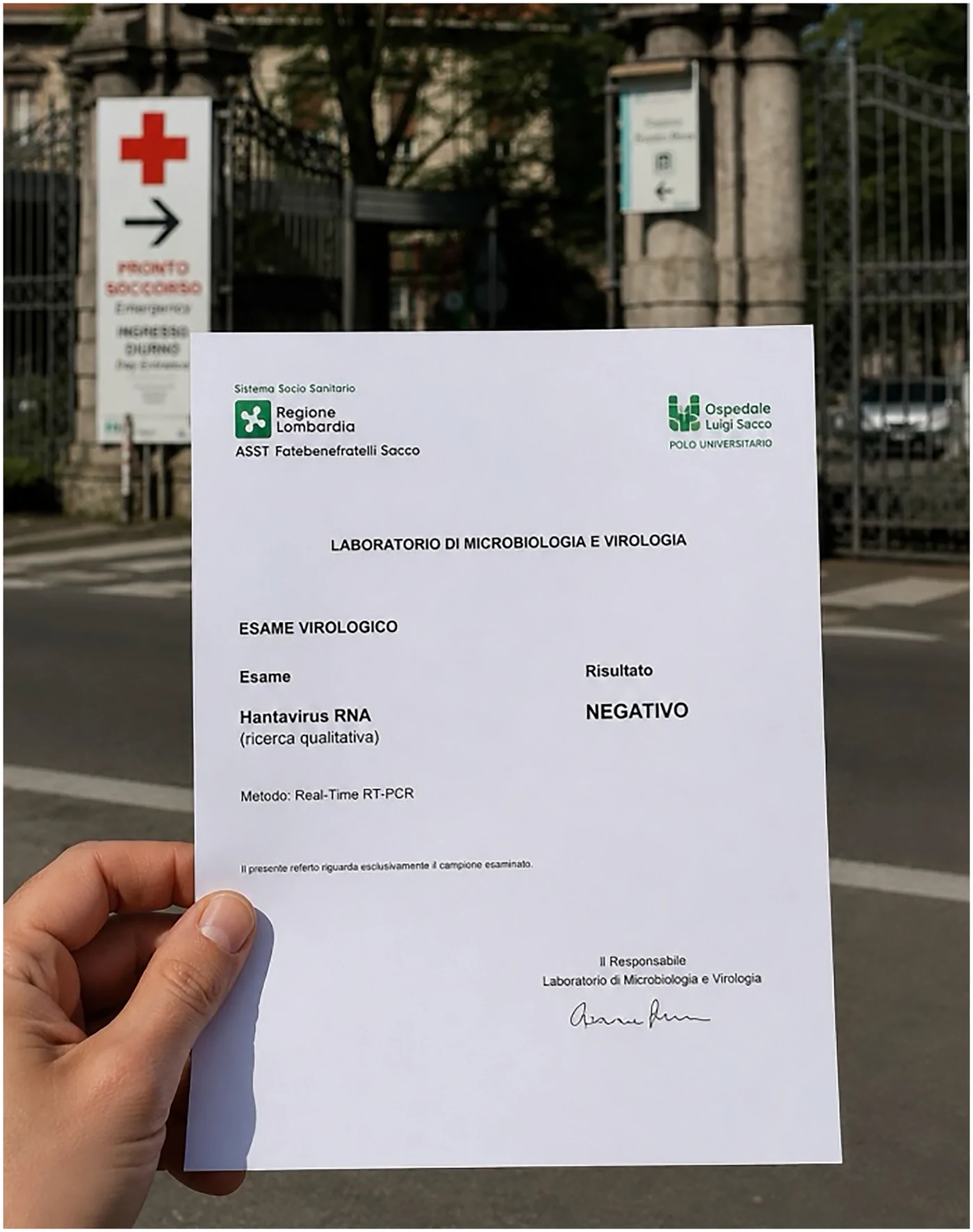
AI-generated ANDV diagnostic report. AI-generated Virology report displaying a negative Hantavirus RNA screening result using Real-Time PCR analysis performed at the Microbiology lab of Ospedale Luigi Sacco, Milan, Italy. Reproduced with permission from “HANTAVIRUS: TURISTA NEGATIVO AL SACCO” by mi_tomorrow.

On May 12, 2026, Italian media outlets reported the isolation and hospitalization at “Luigi Sacco Hospital” in Milan of a contact linked to a confirmed Andes virus (ANDV) case associated with the MV Hondius outbreak reported on May 4, 2026, by the World Health Organization (WHO) (9). One article included an image, disclosed as AI-generated, of a hand holding a document presented as a diagnostic report for ANDV, showing a “NEGATIVE” result (10) (Figure 1).

The alleged hospital laboratory report reproduced the visual identity of “Luigi Sacco Hospital”, potentially increasing its perceived authenticity.

Closer inspection revealed multiple inconsistencies, including inaccurate institutional branding, absence of patient identifiers, atypical microbiological terminology, and reporting features inconsistent with contemporary digitally validated laboratory workflows. The overall visual composition also reflected the hyper-realistic aesthetic typical of contemporary AI-generated images, designed to appear convincing during rapid consumption while remaining fragile under professional scrutiny.

Further articles published during the same week by major Italian newspapers included AI-generated images depicting positive hantavirus tests, Italian authorities screening alleged hantavirus contacts at the airport, and hospital-based infectious disease response activities (11–13). In these last three examples the use of genAI for the creation of these realistic images has not been disclosed.

These episodes may simply reflect an editorial decision to illustrate news articles using synthetic content. However, even when not intentionally deceptive, the circulation of realistic but non-authentic medical-looking documents, samples, and procedures may distort public risk perception, reinforce the impression of clinically verified information, and contribute to misunderstanding during infectious disease (ID) alerts when public attention is heightened.

## Discussion

In recent weeks, increasing media attention regarding hantavirus infections has generated understandable public concern particularly on social media platforms and digital communication channels. Within this context, the online circulation of an alleged hospital laboratory report related to hantavirus testing, reproducing the visual identity of “Luigi Sacco Hospital” in Milan, which is a referral centre for ID and pandemic preparedness, represents an emblematic example of the new challenges introduced by AI-generated medical realism.

The central issue may extend beyond the falsification of a single medical document. The broader concern is the emergence of “synthetic medical realism”: AI-generated content capable of exploiting the symbolic and visual authority traditionally associated with healthcare institutions. Medicine relies heavily on visual trust, and medical images are not only neutral representations of reality but also sociocultural constructions that shape credibility and transmit certainty (14). Laboratory reports, radiological images, pathology slides, and hospital-branded documents are frequently perceived as credible before undergoing verification. As a result, fabricated medical content presented in a convincing-enough medical form may rapidly activate cognitive trust mechanisms among patients, journalists, and even healthcare professionals.

Recent literature increasingly supports these concerns. Zhu et al. demonstrated that ChatGPT integrated with image-generation tools could create convincing blood smears, Western blots, immunofluorescence images, and other experimental biomedical figures, raising substantial concerns regarding scientific integrity and medical misinformation (4). Similarly, Hartung et al. showed that even domain experts failed to reliably distinguish AI-generated histological images from authentic ones, highlighting the growing difficulty of identifying fabricated biomedical content (5). Tordjman et al. recently demonstrated that radiologists struggled to reliably distinguish synthetic radiographs generated by AI systems from authentic imaging studies (6). In their analysis, neither human experts nor AI detection systems consistently identified all deepfake radiological images. Together, these findings suggest that future ID outbreaks may unfold within a profoundly different communication environment compared with COVID-19. The next infodemic may not simply involve false information but highly realistic synthetic medical content capable of visually simulating clinical authenticity.

This phenomenon may have important public-health implications, not limited to isolated episodes of misinformation, but rather including amplification of public anxiety during outbreaks, confusion regarding testing indications, inappropriate utilization of resources, and erosion of trust in healthcare institutions. For example, synthetic representations of hospital diagnostic reports may incorrectly suggest that specialized ID testing is universally accessible or routinely indicated, potentially generating unrealistic public expectations and unnecessary pressure on healthcare services.

The rapid adoption of genAI in medicine may be outpacing the development of adequate governance frameworks. Current regulatory systems may still be poorly equipped to address AI-generated hallucinations, synthetic medical images, pseudo-clinical outputs, and multimodal misinformation (15). The case described here is emblematic. Although the EU Artificial Intelligence Act (16), which requires disclosure of AI-generated or manipulated audiovisual content that could falsely appear authentic (“deepfakes”), represents an important regulatory step, the relevant transparency obligations under Article 50 will apply only from 2 August 2026, almost three months after the incident described here. Moreover, AI-generated medical content may rapidly circulate across jurisdictions and digital platforms beyond the direct scope of EU regulation and enforcement. The Digital Services Act (17) addresses a different regulatory layer by focusing primarily on intermediary services and online platforms rather than news publishers acting as editorial content producers. The incident therefore illustrates a transitional and functional regulatory gap: realistic AI-generated medical-looking content may circulate before the AI Act's dedicated transparency obligations become applicable.

Furthermore, recent analyses have emphasized that only a minority of AI-enabled medical tools have undergone rigorous interventional clinical evaluation before implementation, and many LLM-based systems used in healthcare communication fall outside traditional medical-device regulatory pathways (18, 19).

Several ethical concerns remain unresolved, including limited transparency regarding AI-generated outputs, unclear accountability for misinformation-related harm, propagation of algorithmic bias, and inappropriate delegation of medical authority to non-validated systems (20).

AI-generated medical realism should be considered a potential emerging category of public health risk during ID emergencies.

Therefore, traditional fact-checking approaches alone may prove insufficient in the near future, and public-health institutions, scientific journals, and healthcare providers may increasingly need to confront the challenge of synthetic medical realism. Future preparedness strategies may warrant coordinated action across multiple actor groups. Public health institutions should develop rapid institutional verification systems for suspicious medical content circulating during outbreaks, and establish public communication protocols that anticipate and addressing synthetic medical misinformation before it shapes public risk perception during ID alerts. News media outlets should adopt and enforce explicit editorial standards requiring clear disclosure of AI-generated imagery, particularly when such content depicts or resembles clinical documentation, hospital materials, or outbreak-related medical information as the absence of disclosure, even when unintentional, may distort public perception. Digital platforms should implement scalable monitoring systems capable of detecting and flagging AI-generated health misinformation during outbreak events, and invest in the development and integration of digital watermarking and content-authenticity verification tools to facilitate rapid identification of synthetic medical content. Scientific journals should establish explicit submission guidelines and editorial policies, while regulators could consider expediting the implementation and updating of European regulations on this matter in a sector-specific manner, to promptly close transitional regulatory gaps as illustrated by this case.

In conclusion, the unregulated use of genAI may represent a significant emerging threat to public health communication during infectious disease emergencies, highlighting the urgent need for stronger policies focused on the education, regulation, and monitoring of AI-generated media as a key preparedness strategy.

## Data Availability

The original contributions presented in the study are included in the article/Supplementary Material, further inquiries can be directed to the corresponding author/s.
